# Evaluating the “holiday season effect” of hospital care on the risk of mortality from pulmonary embolism: a nationwide analysis in Taiwan

**DOI:** 10.1038/s41598-021-98845-5

**Published:** 2021-09-29

**Authors:** Duan-Pei Hung, Shu-Man Lin, Peter Pin-Sung Liu, I-Min Su, Jin-Yi Hsu, Ting-Yu Wu, Chu-Chun Lin, Huei-Kai Huang, Ching-Hui Loh

**Affiliations:** 1 Department of Family Medicine, Hualien Tzu Chi Hospital, Buddhist Tzu Chi Medical Foundation, Hualien, Taiwan, ROC; 2 Department of Psychiatry, Taipei Veterans General Hospital, Taipei, Taiwan, ROC; 3 Department of Physical Medicine and Rehabilitation, Hualien Tzu Chi Hospital, Buddhist Tzu Chi Medical Foundation, Hualien, Taiwan, ROC; 4 School of Medicine, Tzu Chi University, Hualien, Taiwan, ROC; 5 Center for Aging and Health, Hualien Tzu Chi Hospital, Buddhist Tzu Chi Medical Foundation, No. 707, Sec. 3, Chung Yang Rd., Hualien, 97002 Taiwan, ROC; 6 Institute of Medical Sciences, Tzu Chi University, Hualien, Taiwan, ROC; 7 Department of Anesthesiology, Hualien Tzu Chi Hospital, Buddhist Tzu Chi Medical Foundation, Hualien, Taiwan, ROC; 8 Department of Orthopedics, Hualien Tzu Chi Hospital, Buddhist Tzu Chi Medical Foundation, Hualien, Taiwan, ROC; 9 Department of Psychiatry, Far Eastern Memorial Hospital, New Taipei City, Taiwan, ROC; 10 Department of Medical Research, Hualien Tzu Chi Hospital, Buddhist Tzu Chi Medical Foundation, No. 707, Sec. 3, Chung Yang Rd., Hualien, 97002 Taiwan, ROC

**Keywords:** Cardiovascular diseases, Cardiology, Health care

## Abstract

We aimed to determine whether hospital admissions during an extended holiday period (Chinese New Year) and weekends were associated with increased mortality risk from pulmonary embolism (PE), compared to admissions on weekdays. We conducted a nationwide retrospective cohort study using Taiwan’s National Health Insurance Research Database. Data of newly diagnosed PEs during the months of January and February from 2001 to 2017 were obtained from patient records and classified into three admission groups: Chinese New Year (≥ 4 consecutive holiday days), weekends, and weekdays. The adjusted odds ratios (aORs) (95% confidence intervals [CIs]) for 7-day and in-hospital mortality were calculated using multivariable logistic regression models. The 7-day and in-hospital mortality risks were higher for patients admitted during the Chinese New Year holiday (10.6% and 18.7%) compared to those admitted on weekends (8.4% and 16.1%) and weekdays (6.6% and 13.8%). These higher mortality risks for holiday admissions compared to weekday admissions were confirmed by multivariable analysis (7-day mortality: aOR = 1.68, 95% CI 1.15–2.44, P = 0.007; in-hospital mortality: aOR = 1.41, 95% CI 1.05–1.90, P = 0.022), with no subgroup effects by sex or age. Hospital admission for PE over an extended holiday period, namely Chinese New Year, was associated with an increased risk of mortality.

## Introduction

Venous thromboembolism, which includes deep vein thrombosis and pulmonary embolism (PE), is the third most common cardiovascular disease, after myocardial infarction and stroke, causing death and disability^1^. The incidence of PE varies among ethnicities and increases with age, with an overall annual incidence rate of 7–115 per 100,000 individuals^2,3^. A previous large-scale study in the United States reported in-hospital and 30-day mortality rates of 7.9% and 9.4%, respectively, in adult patients hospitalized with PE^4^. A large proportion of hospitalized patients are at risk of developing venous thromboembolism. In one study, 42% of medical patients and 64% of surgical patients were at moderate or high risk of venous thromboembolic events^5^. With the upward trend of the risk factors for venous thromboembolism, such as aging and obesity, the incidence of PE is expected to increase.

PE is a critical condition that requires a careful and experienced clinical examination to make a timely diagnosis. Recent developments in the knowledge of PE have provided detailed guidelines for the evaluation and management of PE^6^. Early intervention and diagnosis of PE can be life-saving and cost-effective, as deterioration in respiratory function with PE can lead to right ventricular strain, the requirement for ventilation, and admission to the intensive care unit. Currently, however, prompt evaluation and management of PE is not as widely emphasized as for other conditions, such as stroke or myocardial infarction. Moreover, the negative effect of weekend admissions on the outcomes of various time-sensitive medical conditions, which has been widely described^7–10^, may further impact the outcomes of PE^11–20^. A study by Nanchal et al.^15^ indicated that the time to intervention is a potential mediating factor for the observed weekend effect on the outcomes of PE, which may itself be associated with the reduced number of medical staff on weekends, who often have less practical experience.

In Asia, the Chinese New Year, which is a holiday of ≥ 4 days, can prolong the negative weekend effect on the diagnosis and management of PE. During this holiday period, hospitals in countries that celebrate this holiday usually have a substantial reduction in staffing, with a corresponding shut down of certain wards. However, evidence regarding the negative impact of the weekend effect on PE is limited overall, with varying conclusions presented^11–20^. Furthermore, whether the effect on time-sensitive medical conditions, such as PE, is more evident during consecutive holidays is still unknown. Therefore, the aim of this study was to compare the mortality risk among patients admitted for PE during the Chinese New Year holiday, weekends, and weekdays, in Asia, where this issue has not been sufficiently evaluated.

## Methods

### Data source

Our analysis was based on data from the Taiwan’s National Health Insurance Research Database (NHIRD). This database contains the health data of approximately 25 million individuals enrolled in the National Health Insurance (NHI) program. The NHI program is a universal, single-payer health insurance program in Taiwan, which was founded in 1995 and includes > 99% of the Taiwanese population. Moreover, 97% of the 480 hospitals and 22,000 clinics in Taiwan have a contract with the NHI program. The database includes individual demographic and income data, and the following health data: diagnoses, treatments, and the cost of medical (in- and out-patient) care for hospitals of all levels and medical providers. All individual identifiable information in the NHIRD is removed before release for research purposes and is encrypted under an access policy.

### Ethics declarations

Our study was conducted in accordance with the World Medical Association Declaration of Helsinki: Ethical Principles for Medical Research Involving Human Subjects. The study protocol was approved by the Hualien Tzu Chi Hospital Research Ethics Committee (IRB107-152-C). The requirement for informed consent was waived owing to the retrospective nature of the study, and the use of anonymized NHIRD data.

### Study population

We included all adult patients aged ≥ 20 years who had been admitted to hospital in January or February from 2001 through 2017, and had been diagnosed with new-onset PE (as per the International Classification of Diseases [ICD]-9 code, 415.1 or the ICD-10 code, I26). We excluded patients with missing common demographic information (such as sex and date of birth), those with a previous diagnosis of PE (prior to the index hospitalization), and those with a duration of hospitalization of > 6 months. As our study aimed to evaluate the adult population, we excluded patients aged < 20 years. In Taiwan, a person is legally considered an adult at the age of 20, according to Civil Law. The patient selection process and a brief overview of the demographic information of the excluded patients are summarized in Supplementary Fig. S1 online.

### Definition of independent variables

The independent variable in the analysis was the day of admission, namely during the Chinese New Year holiday (consecutive days of holiday based on the lunar calendar), on a non-holiday weekend (Saturday or Sunday), or on a non-holiday weekday (Monday through Friday). Of note, the exact dates of the Chinese New Year differ each year due to the discrepancy between the solar and lunar calendars; however, it does fall in the months of January and February, as per the Gregorian calendar. For our study, we obtained the annual dates of the Chinese New Year from 2001 to 2017 from Taiwan’s national holiday lists. In order to minimize the variation of seasonal diseases resulting from seasonal variation in mortality, we only extracted hospitalization records for the months of January and February, annually.

### Outcomes

The primary outcomes were the 7-day and in-hospital mortality, defined after admission for PE. The date of death was confirmed by linking patient files in the NHIRD to the National Register of Deaths in Taiwan. The mortality risk was compared between the three admission periods (Chinese New Year holiday, weekends, and weekdays), using the weekday admission as the reference group.

### Covariates and potential confounders

Information on patients’ pre-existing comorbidities was obtained by retrieving individuals’ ICD-9-CM diagnostic codes from in- and out-patient records. Baseline comorbidities were those diagnosed (on an in- or out-patient basis) within the 12-month period preceding the index hospitalization. Charlson Comorbidity Index scores were used to quantify the burden of comorbidities and provide the overall health status, providing a valid index of a patient’s mortality risk^21^. Hospitals in Taiwan are classified into three levels (medical center, regional hospital, and district hospital), as determined by the Joint Commission of Taiwan, based on the quality of care provided, completeness of specialty services, and the number of beds. The level of a hospital and its location determine the services available and resources, both of which may influence disease outcomes. Patient income levels were categorized into the following four intervals for analysis: financially dependent, 15,840–24,999 New Taiwan dollars (TWD), 25,000–39,999 TWD, and ≥ 40,000 TWD. The geographic locations of the participating hospitals were categorized according to the four administrative areas in Taiwan: northern, central, southern, and eastern.

### Statistical analyses

Baseline characteristics were compared between the three admission groups using a chi-squared test for categorical variables and analysis of variance (ANOVA) for continuous variables. A multivariable logistic regression analysis was used to calculate the odds ratios (ORs) for the in-hospital and 7-day mortality, adjusted for all baseline characteristics (Table 1). Sub-analyses stratified by sex and age were also performed. We conducted a test for interaction, using multivariable logistic regression models with the interaction terms “admission type*age group” and “admission type*sex group,” to evaluate whether our study estimates (the effect of admission type on the risk of mortality) differ significantly according to age and sex, respectively^22,23^. Significance was defined as a two-sided probability value < 0.05. Statistical analyses were performed using SAS software (version 9.4; SAS Institute Inc., Cary, NC, USA) and Stata (version 14; Stata Corporation LLC, College Station, TX, USA).

**Table 1:** Baseline characteristics of patients in the three admission groups: Chinese New Year holiday, weekend, and weekday.

Characteristic	Chinese New Year holiday (n = 331)		Weekend (n = 1,065)		Weekday (n = 4,241)		P-value^†^
	n	%	n	%	n	%	
**Age**							0.271
< 65 years	114	34.4	389	36.5	1621	38.2	
≥ 65 years	217	65.6	676	63.5	2620	61.8	
Mean (SD)	68.7	15.1	67.8	16.9	67.1	16.8	0.149
**Sex**							0.444
Male	148	44.7	502	47.1	2041	48.1	
Female	183	55.3	563	52.9	2200	51.9	
**Income level (NTD)**							0.667
Dependent	50	15.1	141	13.2	546	12.9	
15,840–24,999	137	41.4	451	42.4	1723	40.6	
25,000–39,999	87	26.3	263	24.7	1119	26.4	
≥ 40,000	57	17.2	210	19.7	853	20.1	
**Admission hospital level**							0.203
Medical center	173	52.3	512	48.1	1970	46.5	
Regional hospital	138	41.7	462	43.4	1906	44.9	
District hospital	20	6.0	91	8.5	365	8.6	
**Admission hospital area**							0.731
North	137	41.4	465	43.7	1902	44.9	
Central	78	23.6	230	21.6	956	22.5	
South	106	32.0	345	32.4	1286	30.3	
Eastern	10	3.0	25	2.4	97	2.3	
**Charlson Comorbidity Index**^a^							0.518
0	83	25.1	315	29.6	1217	28.7	
1–2	127	38.4	403	37.8	1556	36.7	
≥ 3	121	36.6	347	32.6	1468	34.6	
Mean (SD)	2.4	2.7	2.3	2.7	2.4	2.7	0.433
**Comorbidities**							
Diabetes mellitus	104	31.4	237	22.3	1023	24.1	0.003
Hypertension	164	49.6	538	50.5	2083	49.1	0.715
COPD	60	18.1	189	17.8	835	19.7	0.310
Heart failure	53	16.0	161	15.1	603	14.2	0.546
Coronary artery disease	75	22.7	235	22.1	914	21.6	0.853
Chronic kidney disease	31	9.4	101	9.5	442	10.4	0.584
Cirrhosis	26	7.9	80	7.5	289	6.8	0.600
Stroke	54	16.3	173	16.2	655	15.4	0.767
Dementia	21	6.3	71	6.7	254	6.0	0.703
Malignancy	57	17.2	195	18.3	788	18.6	0.821

*COPD* chronic obstructive pulmonary disease, *n* number, *NTD* New Taiwan dollars, *SD* standard deviation.

^†^Categorical variables were compared using the chi-squared test and continuous variables using analysis of variance.

^a^Calculated without including scores for age.

## Results

### Characteristics of the study group

The characteristics of our study group are summarized in Table 1. Our study group included 5637 patients admitted to participating hospitals in January or February from 2001 to 2017 with a primary diagnosis of PE. The mean age of the study population was 67 years, with women accounting for 52%. Except for diabetes mellitus, there were no significant differences in baseline characteristics across the three admission groups (Table 1).

### In-hospital and 7-day mortality

The 7-day mortality rates for PE were 10.6%, 8.4%, and 6.6% for the Chinese New Year holiday, weekend, and weekday admission groups, respectively. The in-hospital mortality rates were 18.7%, 16.1%, and 13.8% for the three admission groups, respectively (Table 2). In both univariable and multivariable logistic regression models, the 7-day and in-hospital mortality risks were higher for patients admitted during the Chinese New Year holiday compared to those admitted on weekdays. After adjusting for confounders, admission during the Chinese New Year holiday was associated with a 68% higher 7-day and a 41% higher in-hospital mortality risk, with significant adjusted ORs (aORs): 7-day mortality (aOR = 1.68, 95% confidence interval [CI] 1.15–2.44, P = 0.007) and in-hospital mortality (aOR = 1.41, 95% CI 1.05–1.90, P = 0.022). The 7-day mortality (aOR = 1.29, 95% CI 1.01–1.66, P = 0.044) and in-hospital mortality (aOR = 1.21, 95% CI 1.00–1.46, P = 0.052) risks were also higher for patients admitted on weekends compared to those admitted on weekdays, although the difference was only borderline significant (Table 2).

**Table 2:** Risk of mortality from pulmonary embolism between the three admission groups: Chinese New Year holiday, weekend, and weekday.

Outcome	Admission type	n	Death	Univariable model			Multivariable model^a^		
			n (%)	OR	95% CI	P-value	aOR	95% CI	P-value
7-day mortality	Chinese New Year	331	35 (10.6)	1.67	1.15–2.41	0.007	1.68	1.15–2.44	0.007
	Weekend	1065	89 (8.4)	1.29	1.00–1.65	0.048	1.29	1.01–1.66	0.044
	Weekday	4241	281 (6.6)	1	Ref.	Ref.	1	Ref.	Ref.
In-hospital mortality	Chinese New Year	331	62 (18.7)	1.44	1.08–1.92	0.014	1.41	1.05–1.90	0.022
	Weekend	1065	171 (16.1)	1.19	0.99–1.44	0.062	1.21	1.00–1.46	0.052
	Weekday	4241	586 (13.8)	1	Ref.	Ref.	1	Ref.	Ref.

*aOR* adjusted odds ratio, *CI* confidence interval, *n* number, *OR* odds ratio, *Ref.* reference.

^a^Multivariable logistic regression model with adjustments for all covariates listed in Table 1.

### Sex- and age-stratified analyses and interaction tests

The sex- and age-stratified analyses identified a consistent trend of higher mortality for patients admitted during the Chinese New Year holiday compared to weekday admissions, regardless of sex or age (Table 3), although not all comparisons were significant. The interaction tests revealed that the effect of Chinese New Year holiday admission on the risk of mortality was not significantly modified by sex or age. Similarly, there was no evidence of sex- and age-specific effects on the risk of mortality for patients admitted on weekends compared to those admitted on weekdays.

**Table 3:** Sex- and age-stratified analyses of the risk of mortality from pulmonary embolism between the three admissions groups: Chinese New Year holiday, weekend, and weekday.

Outcome	Stratification	Admission type	n	Death	Multivariable model^a^			Interaction test	
				n (%)	aOR	95% CI	P-value	New year^b^	Weekend^c^
7-day mortality	Male	Chinese New Year	148	18 (12.2)	2.11	1.24–3.60	0.006	0.333	0.792
		Weekend	502	42 (8.4)	1.35	0.94–1.95	0.108		
		Weekday	2041	131 (6.4)	1	Ref.	Ref.		
	Female	Chinese New Year	183	17 (9.3)	1.40	0.82–2.38	0.217		
		Weekend	563	47 (8.4)	1.24	0.88–1.75	0.223		
		Weekday	2200	150 (6.8)	1	Ref.	Ref.		
	Age < 65 years	Chinese New Year	114	12 (10.5)	1.99	1.04–3.81	0.039	0.505	0.124
		Weekend	389	35 (9.0)	1.69	1.12–2.55	0.013		
		Weekday	1621	91 (5.6)	1	Ref.	Ref.		
	Age ≥ 65 years	Chinese New Year	217	23 (10.6)	1.55	0.98–2.45	0.064		
		Weekend	676	54 (8.0)	1.12	0.82–1.54	0.471		
		Weekday	2620	190 (7.3)	1	Ref.	Ref.		
In-hospital mortality	Male	Chinese New Year	148	32 (21.6)	1.45	0.95–2.22	0.082	0.788	0.880
		Weekend	502	91 (18.1)	1.27	0.97–1.65	0.078		
		Weekday	2041	315 (15.4)	1	Ref.	Ref.		
	Female	Chinese New Year	183	30 (16.4)	1.36	0.89–2.08	0.153		
		Weekend	563	80 (14.2)	1.16	0.88–1.53	0.295		
		Weekday	2200	271 (12.3)	1	Ref.	Ref.		
	Age < 65 years	Chinese New Year	114	21 (18.4)	1.70	1.00–2.89	0.049	0.738	0.211
		Weekend	389	50 (12.9)	1.02	0.72–1.44	0.919		
		Weekday	1621	208 (12.8)	1	Ref.	Ref.		
	Age ≥ 65 years	Chinese New Year	217	41 (18.9)	1.34	0.93–1.92	0.117		
		Weekend	676	121 (17.9)	1.31	1.04–1.65	0.020		
		Weekday	2620	378 (14.4)	1	Ref.	Ref.		

*aOR* adjusted odds ratio, *CI* confidence interval, *n* number, *Ref.* reference.

^a^Multivariable logistic regression model with adjustments for all covariates listed in Table 1.

^b^The interaction test was performed to determine whether the association between the Chinese New Year holiday admission (*vs*. weekday admission) and the risk of mortality differed significantly according to age and sex.

^c^The interaction test was performed to determine whether the association between weekend admission (vs. weekday admission) and the risk of mortality differed significantly according to age and sex.

## Discussion

Using a nationwide database, we found a “holiday season effect” on PE, with patients admitted with PE during the Chinese New Year holiday having a 68% higher risk of 7-day mortality, and a 41% higher risk of in-hospital mortality, compared to those admitted with PE on weekdays. We also identified a similar trend towards a higher mortality risk for patients with PE who were admitted on weekends compared to weekdays, although this “weekend effect” was only borderline significant. To our knowledge, this is the first study to specifically explore the effect of hospital admission during consecutive national holidays on the mortality risk of PE.

Prior studies have reported worse outcomes for patients who were admitted for time-sensitive conditions on weekends, which constitutes the so-called “weekend effect”. Nanchal et al*.*^16^ reported an increased risk of mortality from PE for patients admitted on weekends, compared to those admitted on weekdays (OR = 1.17, 95% CI 1.11–1.22). They further explored the time to thrombolytic therapy or inferior vena cava filter placement between weekend and weekday admission groups, with longer time to treatment reported on weekends, which may explain the worse patient-related outcomes. Longer holidays may be associated with a greater impact through a similar pathway. A previous study demonstrated that consecutive days of holiday have a significant impact on the risk of mortality for patients with stroke^24^. Our present study further demonstrated the effect of an extended holiday period on the risk of mortality for patients with PE.

We also performed a subgroup analysis of patients admitted to hospital within 5 days prior to the Chinese New Year holiday (termed the pre-holiday group), as shown in Supplementary Table S1 online. This group may also be influenced by the “holiday season effect”, if PE occurred during hospitalization and very close to the Chinese New Year’s Eve. However, we found that the pre-holiday group did not have a higher risk of mortality than the weekend or weekday groups. Nevertheless, the Chinese New Year holiday group still had a higher risk of mortality than the pre-holiday group (see Supplementary Table S1 online). As noted above, PE is a critical condition that requires timely management. It was possible that most “holiday season effect” determinants, such as the timing of PE events, PE diagnosis, and time to treatment, occurred in the pre-holiday period (before the Chinese New Year), when medical resources, staffing levels, and patient load were more similar to those on normal working days. These findings further support the hypothesis that patients admitted to hospital on consecutive holiday days (namely the Chinese New Year holiday) tend to have higher risk of mortality, compared to those admitted on weekdays, weekends, or during the pre-holiday period. In addition, we observed a decrease in PE mortality over the years in Taiwan, which may be attributable to better management of PE in recent years (see Supplementary Fig. S2 online).

Ideally, hospitals should have the capacity to provide the same level of care, regardless of the day of the week, hour of the day, or holidays. However, in the real world, holidays are associated with a reduction in medical and non-medical staff^25^. Consequently, physicians working during holidays are responsible for a larger patient load, compared to coverage during non-holidays and weekdays. As such, service providers are less familiar with hospitalized patients during these periods of high coverage. Moreover, with a decrease in subspecialty coverage during weekends and holidays, timely diagnosis and advanced diagnostic approaches are limited. All of these factors may contribute to the unavailability of timely intervention, which can have a negative impact on the patient outcomes of PE.

Although our study could not infer a causal relationship of the longer “holiday season effect” directly on the worse outcome of PE, we did show that the previously reported “weekend effect” on patient mortality risk was enhanced during the prolonged holiday period for the Chinese New Year in Taiwan. Knowledge of this “holiday season effect” is important to increase awareness among medical personnel to pursue advanced diagnostic modalities for patients with suspected PE, such as echocardiography and computed tomography pulmonary angiography, to lower the risk of mortality. Our findings also have implications for policy makers, highlighting the need to develop and implement strategies to maintain the quality of hospital medical care during extended holiday periods.

The limitations of our study need to be acknowledged and addressed in future studies. First, our analysis did not include detailed clinical data, which may influence the outcomes of PE, including body mass index, lifestyle, smoking, and alcoholism, and did not include imaging reports and laboratory tests. Further, we could not determine the severity of PE. Therefore, unknown or unmeasured confounders may have existed, leading to bias in this observational study. Second, as we used claims-based data, process indicators, such as the time to anti-coagulation therapy initiation, time to achieve target levels, and time from symptom onset to diagnosis and treatment, could not be evaluated. Information on time-specific hospital workforce, availability of interventions, and diagnostic and therapeutic procedure capacity during a period of extended holiday are also warranted to establish real causality between the “holiday season effect” and worse outcomes of time-sensitive medical conditions, such as PE.

In conclusion, this nationwide, retrospective cohort study found that hospital admission for PE during the Chinese New Year holiday was associated with a 68% higher risk of 7-day mortality, and a 41% higher risk of in-hospital mortality, compared to weekday admissions. Our findings also highlight the need to develop and implement strategies to maintain the quality of hospital medical care during extended holiday periods. Further studies are needed to explore the exact underlying mechanism and determine causality.

## Supplementary Information

Supplementary Information.

## Data Availability

The dataset used in this study is managed by the Taiwan Ministry of Health and Welfare and, thus, cannot be made publicly available. Researchers wishing to access this dataset can submit a formal application to the Taiwan Ministry of Health and Welfare (No. 488, Sec. 6, Zhongxiao E Rd., Nangang District, Taipei City 115, Taiwan; https://dep.mohw.gov.tw/DOS/cp-2516-59203-113.html) to request access.
