# Supplementary material for: Evaluating the “holiday season effect” of hospital care on the risk of mortality from pulmonary embolism: a nationwide analysis in Taiwan
Source: Sci Rep. 2021 Sep 29;11:19376. doi: 10.1038/s41598-021-98845-5 (PMC8481409; doi:10.1038/s41598-021-98845-5)
Supplement: Supplementary file 1 — Supplementary Information. [file 41598_2021_98845_MOESM1_ESM.pdf]

Supplemental Materials

Evaluating the "holiday season effect" of hospital care on the risk of mortality from pulmonary embolism: a nationwide analysis in Taiwan

Duan-Pei Hung, M.D.; Shu-Man Lin, M.D.; Peter Pin-Sung Liu, M.S.; I-Min Su, M.D.; Jin-Yi Hsu, M.D.; Ting-Yu Wu, M.D.; Chu-Chun Lin, M.D.; Huei-Kai Huang, M.D.; and Ching-Hui Loh, M.D., Dr.P.H.

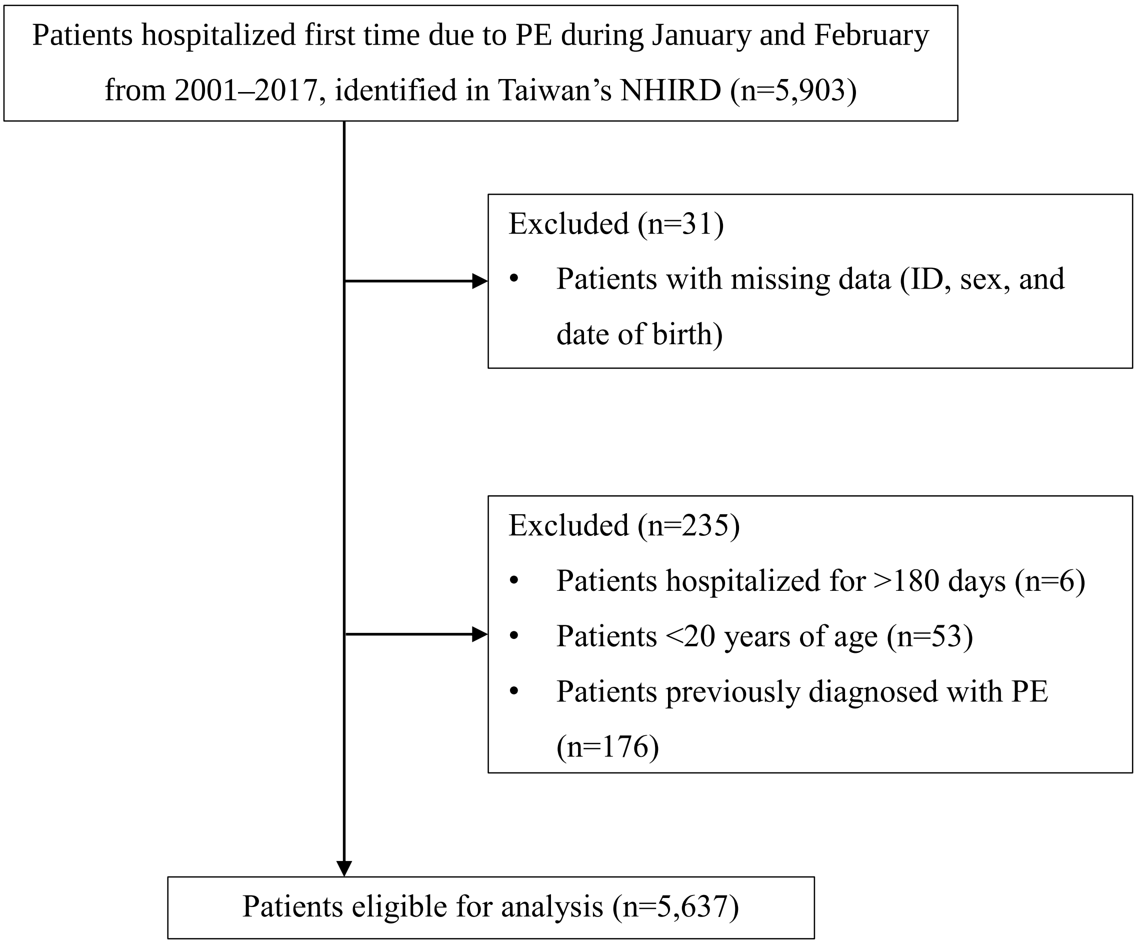

Supplementary Figure S1. Flowchart of patient selection.

In the excluded group of 31 patients with missing data (ID, sex, and date of birth), the in-hospital and 7-day mortality rates were 3.2% (n=1) and 0.0% (n=0), respectively. In the excluded group of 235 patients (those hospitalized for >180 days, <20 years of age, or previously diagnosed with PE) (mean age, 55.7 years; female, 55.6%), the in-hospital and 7-day mortality rates were 3.8% (n=9) and 1.3% (n=3), respectively.

Abbreviations: ID, Identification; NHIRD, National Health Insurance Research Database; PE, pulmonary embolism.

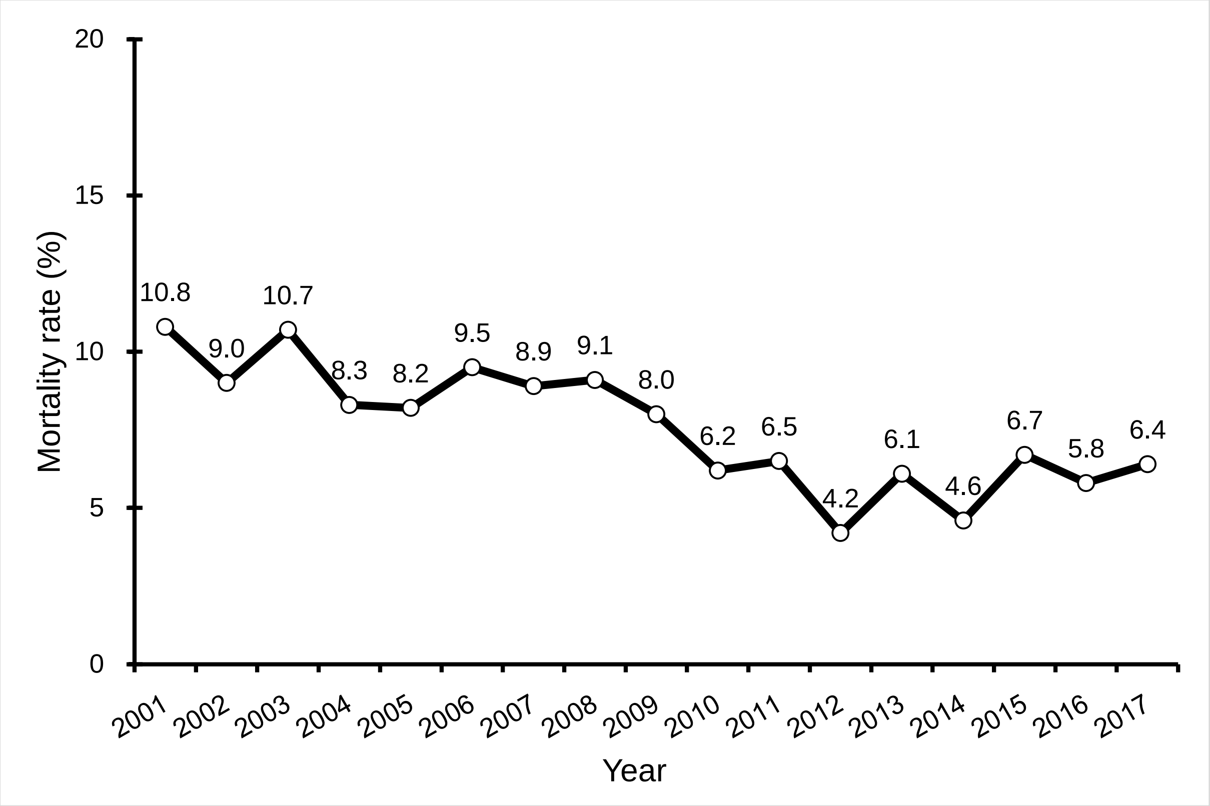

Supplementary Figure S2. Overall annual 7-day mortality risk from pulmonary embolism in Taiwan.

Supplementary Table S1. Risk of mortality from PE in patients admitted during the pre-holiday* period compared with those admitted during weekdays, weekends, and the Chinese New Year holiday

Outcome	Comparison	Multivariable model†		
		aOR	95% CI	P-value
7-day mortality	Pre-holiday *vs*. weekdays	0.83	0.53–1.31	0.427
	Pre-holiday *vs*. weekends	0.63	0.39–1.03	0.066
	Chinese New Year *vs*. pre-holiday	1.98	1.11–3.54	0.021
In-hospital mortality	Pre-holiday *vs*. weekdays	0.92	0.67–1.25	0.597
	Pre-holiday *vs*. weekends	0.75	0.53–1.06	0.100
	Chinese New Year *vs*. pre-holiday	1.50	0.98–2.32	0.064

* Pre-holiday group defined as patients admitted for PE within 5 days prior to the Chinese New Year holiday
† Multivariable logistic regression model adjusted for all covariates listed in Table 1.
Abbreviations: aOR, adjusted odds ratio; CI, confidence interval; PE, pulmonary embolism.
